# Feasibility of large-scale deployment of multiple wearable sensors in Parkinson's disease

**DOI:** 10.1371/journal.pone.0189161

**Published:** 2017-12-20

**Authors:** Ana Lígia Silva de Lima, Tim Hahn, Luc J. W. Evers, Nienke M. de Vries, Eli Cohen, Michal Afek, Lauren Bataille, Margaret Daeschler, Kasper Claes, Babak Boroojerdi, Dolors Terricabras, Max A. Little, Heribert Baldus, Bastiaan R. Bloem, Marjan J. Faber

**Affiliations:** 1 Department of Neurology, Donders Institute for Brain, Cognition and Behaviour, Radboud University Medical Center, Nijmegen, The Netherlands; 2 CAPES Foundation, Ministry of Education of Brazil, Brasília/DF, Brazil; 3 Intel, Advanced Analytics, Tel Aviv, Israel; 4 The Michael J Fox Foundation for Parkinson’s Research, New York, United States of America; 5 UCB Biopharma, Brussels, Belgium; 6 Aston University, Birmingham, United Kingdom; 7 Media Lab, Massachusetts Institute of Technology, Cambridge, United States of America; 8 Philips Research, Department Personal Health, Eindhoven, the Netherlands; 9 Radboud University Medical Center, Radboud Institute for Health Sciences, Scientific Center for Quality of Healthcare, Nijmegen, the Netherlands; Hospital General Dr. Manuel Gea Gonzalez, MEXICO

## Abstract

Wearable devices can capture objective day-to-day data about Parkinson’s Disease (PD). This study aims to assess the feasibility of implementing wearable technology to collect data from multiple sensors during the daily lives of PD patients. The Parkinson@home study is an observational, two-cohort (North America, NAM; The Netherlands, NL) study. To recruit participants, different strategies were used between sites. Main enrolment criteria were self-reported diagnosis of PD, possession of a smartphone and age≥18 years. Participants used the Fox Wearable Companion app on a smartwatch and smartphone for a minimum of 6 weeks (NAM) or 13 weeks (NL). Sensor-derived measures estimated information about movement. Additionally, medication intake and symptoms were collected via self-reports in the app. A total of 953 participants were included (NL: 304, NAM: 649). Enrolment rate was 88% in the NL (n = 304) and 51% (n = 649) in NAM. Overall, 84% (n = 805) of participants contributed sensor data. Participants were compliant for 68% (16.3 hours/participant/day) of the study period in NL and for 62% (14.8 hours/participant/day) in NAM. Daily accelerometer data collection decreased 23% in the NL after 13 weeks, and 27% in NAM after 6 weeks. Data contribution was not affected by demographics, clinical characteristics or attitude towards technology, but was by the platform usability score in the NL (*χ*^2^ (2) = 32.014, p<0.001), and self-reported depression in NAM (*χ*^2^(2) = 6.397, p = .04). The Parkinson@home study shows that it is feasible to collect objective data using multiple wearable sensors in PD during daily life in a large cohort.

## Introduction

Parkinson’s Disease (PD) is a common neurodegenerative disease in which patients experience both motor and non-motor symptoms[1]. Treatment is primarily based on the management of symptoms by increasing dopamine levels through pharmacological therapy or surgery[2, 3]. Additionally, non-pharmacological therapies, such as physiotherapy, occupational therapy or speech therapy, are available to support patients[4].

Although good results in the management of motor symptoms have been achieved, particularly in the early stages of the disease[5, 6], two major problems hamper long-term treatment. First, current pharmacological therapy is successful for a limited period. In the long term, most patients develop unmanageable motor complications that can lead to worsening of quality of life[7]. Second, evaluation of day-to-day variations in PD symptoms is difficult when relying solely upon periodic consultations by clinicians[8]. Therefore, more detailed, objective and reliable measures during daily living could potentially improve the management of PD.

Wearable sensors have been used to assess PD-related symptoms continuously and longitudinally during daily living[9–12]. Wearables may provide greater insight into a patient’s disease status, allowing patients to self-manage their symptoms and monitor medication responses[13–18]. Furthermore, wearable sensor data may improve our scientific understanding of disease progression by showing changes in motor and non-motor symptoms over time, furthering the development of digital biomarkers for disease progression[19].

While the potential value of wearable sensors for disease management and research are increasingly becoming clear, various critical aspects of feasibility remain to be determined. Only a few studies have rigorously investigated the feasibility and acceptability of using a wearable platform comprising a smartphone in combination to a smartwatch. Moreover, these prior findings remained limited by the small sample sizes (biggest sample thus far: 40 PD patients) [9, 13, 17, 18]. Therefore, we aimed to investigate the feasibility of using a wearable platform in a much larger sample of PD patients, with a focus on recruitment success, attrition rates, user compliance and system usability.

## Methods

Between August/2015 and November/2016, a total of 953 PD patients from two cohorts (n = 304 in The Netherlands (NL) and n = 649 PD in North America (Unites States and Canada—NAM) participated in the Parkinson@Home feasibility study. To investigate the feasibility of the technology in different contexts, both cohorts used the same wearable platform, but had distinct strategies for recruitment, retention and study period. These topics are described separately (overview in Table 1).

**Table 1:** Study design and procedure overview at the two cohorts.

		The Netherlands	North America
Recruitment strategies	Through Internet communities	✓	✓
	Through support groups	✓	-
	Through physiotherapists	✓	-
Enrolment criteria	≤ 30 years old	✓	-
	Dutch resident	✓	-
	Smartphone using Android OS version 4.2 or higher	✓	✓
	Self-reported PD	✓	✓
	≤ 18 years old	-	✓
	Registered for Fox Insight study	-	✓
	English-speaking Canadian or United States resident	-	✓
Exclusion criteria	None	✓	✓
Consent process	Informative email	✓	✓
	Online digital consent form	✓	✓
Study kit	Pebble smartwatch	✓	✓
	Installation guide	✓	✓
	User manuals	✓	✓
Clinical evaluations	Assessment by physical therapist	✓	-
	Fox Insight online self-assessment surveys	-	✓
Study duration	Minimum of 6 weeks	-	✓
	Minimum of 13 weeks	✓	-
Instruction for device usage	Minimum of 5 hours a day	-	✓
	24 hours, 7 days a week	✓	-
Support model	Call-center during working hours	✓	✓
	Technical support calls for non-data contributors	✓	-
	Support emails for non-contributors	-	✓
Usability questionnaire	-	✓	-

### Study design and population

#### The NL cohort

The population and study design applied in the NL are described in detail elsewhere.[20] In short, participants were recruited from support groups, internet communities and through physiotherapists specialized in treating PD patients. Enrolment criteria were: (1) ≥30 years of age, (2) possession of a smartphone using an Android OS version ≥ 4.2 and (3) self-reported diagnosis of PD. No exclusion criteria were applied beyond enrolment criteria.

All enrolled participants received a single medical examination, based on the “Parkinson's Progression Markers Initiative” (PPMI)[21]. This included the full MDS-UPDRS[22], the Montreal Cognitive Assessment (MoCA)[23], and the Modified Schwab and England Activities of Daily Living Scale.[24] The medical examination was performed by specially trained physiotherapists who are members of ParkinsonNet[25], a Dutch network of health professionals specialised in PD management. At the end of the 13-weeks study period, all enrolled participants evaluated the usability of the system through the System Usability Scale (SUS)[26, 27], and were enquired about ability to use a smartphone (see APPENDIX). Finally, participants had the option to continue using the platform or return the Pebble smartwatch.

#### The NAM cohort

Study recruitment for the NAM cohort was entirely virtual through direct emails to subjects participating in the “Fox Insight online study”, Facebook advertisements to targeted populations, and advertisements on Fox Trial Finder, a clinical trial matching tool for people with PD.[28] Additional to the NL, the following enrolment criteria were applied: (1) ≥18 years of age and (2) participation in the Fox Insight Online Study[29] (Table 1).

In order to enrol, interested participants had to first register in the Fox Insight study (if they had not done so already). Through Fox Insight, each participant completed online surveys about demographics, medical history, cognition, physical activity, symptoms and PD related medications and surgeries. Once enrolled in the Fox Insight, participants were eligible to register for the NAM cohort of the Parkinson@Home study on a separate webpage. These users completed an online enrolment form which was reviewed by the study team to determine eligibility. All study registrants received an email confirming their eligibility or non-eligibility. After finishing the 6-weeks study period, participants had the option to continue using the platform.

### Wearable platform

The Intel^®^ Pharma Analytics Platform used has been described in detail elsewhere.[20, 30] Briefly, it consists of the Fox Wearable Companion app, used on both a smartwatch and smartphone, and a cloud environment. In this study, a Pebble smartwatch, was used together with the patients’ Android phones. 50 Hz accelerometer data were collected continuously from the smartwatch and streamed to the smartphone.

**Fig 1:**
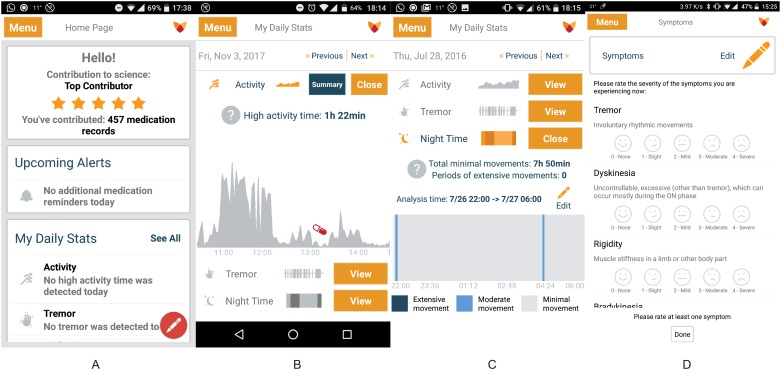
(a) Fox Wearable Companion app main screen; (b) Fox Wearable Companion app activity graph; (c) Fox Wearable Companion app movement during sleep graph; (d) Fox Wearable Companion app symptom self-reports. “Reprinted from [Intel and Michael J Fox Foundation] under a CC BY license, with permission from [INTEL^®^], original copyright [2017].

Sensor analysis algorithms are applied to the aggregated (30 second interval) smartwatch accelerometer data in the app to estimate outcomes (i.e. levels of activity, tremor and movement during sleep). These estimated quantities are transmitted, via Wi-Fi or mobile data, to a cloud environment. They are also presented to the user by graphs and summary reports within the app. Additionally, users are able to set medication reminders, report actual medication intake and rate their symptoms (e.g. tremor, dyskinesia, rigidity, bradykinesia) within the mobile app (Fig 1). Both estimated outcomes and patients reported outcomes (PROs) are stored in the cloud environment.

### Study procedures at both cohorts

Participants from both cohorts provided electronic consent and received a research kit containing a Pebble smartwatch, an installation guide and user manuals. Next, participants installed the Fox Wearable Companion App on their devices and were asked to wear the smartwatch and keep their smartphone with them as much as possible on either a 24/7 basis for 13-weeks study period (NL) or for a minimum of 5 hours a day, 7 days a week, for a 6-weeks study period (NAM). Additionally, participants reported their medication intake (i.e medication name and doses) and PD symptom severity using the app. A helpline was available during the study period for technical support. Support calls or emails were sent to participants from whom data were not collected for more than seven consecutive days.

### Outcome definitions and statistical analysis

Feasibility assessment included recruitment, attrition, compliance and system usability. Recruitment success was analysed by (1) the total number of enrolled participants and (2) the number of eligible registrants that did not complete the informed consent. Compliance, similar to previous studies[31, 32], was calculated as the median percentage of the study period where accelerometer data were collected. Attrition rate, based upon Eysenbach et al. [33], were measured by (1) decrease in the daily percentage of collected accelerometer data during each study period and (2) decrease in the number of participants contributing accelerometer data. Finally, system usability was measured by the median total score on the System Usability Scale.

We investigated the relationship between self-reported demographics, clinical data, ability to use a smartphone (S1 File), System Usability score and the percentage of accelerometer data collected to identify factors that influence compliance levels. Participant demographic and clinical characteristics were grouped into categories either following previously described literature (presence of depression[34]; presence of cognitive impairment[23]) or by convenience (age; educational level: a measure of the last completed level of education where low education was equal to high school or lower levels, middle education was equal to bachelor, and high education was equal to master or higher levels; Hoehn & Yahr stage and Modified Schwab and England scale). Because compliance was not normally distributed, the median and quartiles were used to divide participants into three compliance groups (low, middle and high). The first quartile was the cut-off for the low compliant group and third quartile for the high compliant group. Depending on the distribution of other variables in the analysis, either Chi-square, Fisher’s Exact Test or Kruskal-Wallis were used to investigate significant differences between compliance groups considering demographics, clinical characteristics, ability to use a smartphone and System Usability score.

### Ethics standards

This study was conducted in compliance with the Ethical Principles for Medical Research Involving Human Subjects, as defined in the Declaration of Helsinki. The study protocol and communication materials were approved by the local ethics committee (NL: CMO Arnhem-Nijmegen; NL53034.091.15; NAM: New England IRB: 15–046).

## Results

### Recruitment and sample characteristics

In the NL cohort, 347 eligible PD patients were invited to participate. Among those invited, 43 refused to participate. The main refusal reasons were “Study protocol seems too burdensome” (44%, n = 19), followed by “Personal circumstances” (33%, n = 14). A total of 304 patients (enrolment rate = 88%) were enrolled.

In the NAM cohort, from the 866 participants of the Fox Insight study who received a direct invitation to participate, 306 were enrolled (6% were ineligible). 344 additional participants were included from the remaining recruitment channels, with varied ineligibility rates. A total of 649 registrants (enrolment rate = 51%) were enrolled.

In both cohorts, 953 participants were enrolled. From them, 805 were data contributors (participants that contributed at least one accelerometer data point during study period). Analysis of the demographic characteristics of both cohorts showed that, in comparison to NA, the NL cohort presented more men (χ2 (1) = 9.5146, p<0.01); older (χ2 (2) = 16.435, p = 0.001) and higher educated (χ2 (2) = 25.270, p<0.001) PD included participants. The characteristics of all participants are presented in Table 2.

**Table 2:** Demographic and disease related characteristics of participants.

		The Netherlands			North America			Total	
		*Data contributors* (n = 291)*	*Non-compliant* (n = 13)*	*p-value*	*Data contributors* (n = 514)*	*Non-compliant* (n = 135)*	*p-value*	*Data contributors* (n = 805)*	*Non-compliant* (n = 148)*
Sex	Men	168 (65%)	-	NA	260 (53%)	25 (51%)	.763	454 (58%)	25 (51%)
Age	≤50	29 (10%)	1 (8%)	.523	101 (21%)	11 (22%)	.676	130 (17%)	12 (19%)
	51–69	207 (72%)	8 (61%)		310 (63%)	28 (57%)		517 (66%)	36 (58%)
	≥70	53 (18%)	4 (31%)		81 (16%)	10 (20%)		134 (17%)	14 (23%)
Education Level	Low	51 (20%)	NA	NA	168 (35%)	21 (44%)	.477	219 (30%)	21 (44%)
	Middle	103 (40%)	NA	NA	187 (39%)	16 (33%)		290 (39%)	16 (33%)
	High	101 (40%)	NA	NA	126 (26%)	11 (22%)		227 (31%)	11 (22%)
Disease severity	0/1	73 (30%)	1 (100%)	NA	235 (49%)	21 (47%)	.580	308 (43%)	22 (48%)
	2	127 (53%)	0		134 (28%)	10 (22%)		261 (36%)	10 (22%)
	3	34 (14%)	0		98 (20%)	13 (29%)		132 (18%)	13 (29%)
	4/5	6 (2%)	0		13 (3%)	1 (2%)		19 (3%)	1 (2%)
Depression^1^	No	238 (97%)	-	NA	346 (70%)	26 (53%)	.013	584 (79%)	26 (53%)
Cognitive impairment^2^	No (>26)	124 (53%)	1 (100%)	NA	NA	NA	NA	124 (53%)	1 (100%)
Independency level^3^	≤70	36 (15%)	0	NA	NA	NA	NA	36 (15%)	0
	71–80	51 (22%)	0		NA	NA		51 (22%)	0
	81–90	109 (46%)	1 (100%)		NA	NA		109 (46%)	1 (100%)
	≥91	41 (17%)	0		NA	NA		41 (17%)	0
How easy is for you to use a smartphone?	Very easy	59(22%)	0	.211	NA	NA	NA	59(22%)	0
	Easy	117(44%)	3(50%)		NA	NA		117(44%)	3(50%)
	Neither easy nor difficult	64(22%)	1 (17%)		NA	NA		64(22%)	1 (17%)
	Difficult	20(7%)	2 (33%)		NA	NA		20(7%)	2 (33%)
	Very difficult	6(2%)			NA	NA		6(2%)	
MDS-UPDRS (Median)		52.5 (QR 35–69)	22	-	NA	NA		52.5 (QR 35–69)	22

*Number of missing values differed across variables; only valid percentages are reported.

SES: Socioeconomic status; Disease severity: assessed with Hoehn and Yarh stages at the NL cohort and estimated from self-reported at NAM cohort; MDS-UPDRS: Movement Disorders Society—Unified Parkinson’s Disease Rating Scale.

^1^-Depression was assessed with the Geriatric Depression Scale at NL and self-reported on NAM site;

^2^-Cognitive impairment was assessed using the Montreal Cognitive Assessment,

^3^-Independency level was measured by Modified Schwab and England Activities of Daily Living Scale.

NA = not assessed. QR: 1^st^ and 3^rd^ quartiles.

### Technical support to participants

In both cohorts, the helpdesk consisted of two research assistants, available for 20 hours (NL) and 40 hours (NAM) per week. The actual workload was dependent on: (1) the number of participants simultaneously enrolled in the trial; and (2) the occurrence of bugs in the app or server downtime. The most frequent and time-consuming problems were: (1) Bluetooth disconnection between the smartwatch and the smartphone and (2) questions regarding the medication report, especially in the first weeks of participation.

### Compliance

**Fig 2:**
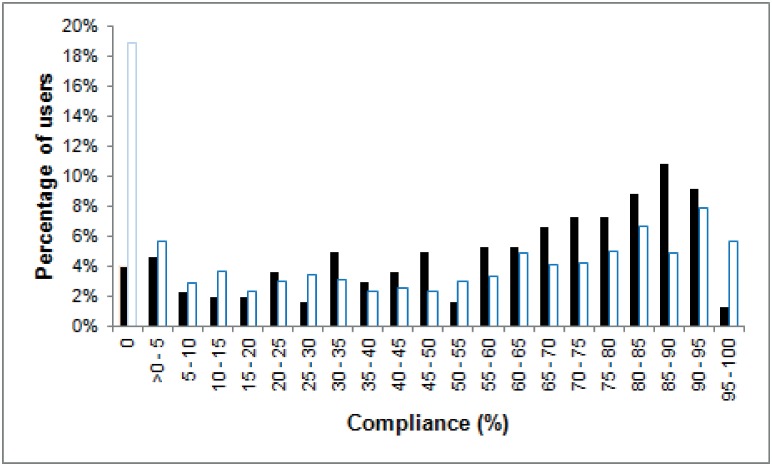
Distribution of compliance among all enrolled participants in the NL (n = 304-black) and NAM (n = 649-white) study cohorts.

Among both cohorts, 85% (n = 805 of 953 enrolled) of participants were data contributors. In the NL, 291 data-contributors collected data for a median of 1,478 hours each in the 13-weeks, with quartile ranges (1^st^ and 3^rd^ QR) of 888 to 1,827 hours. In NAM, 514 data contributors collected a median of 621 hours (1^st^ QR: 286 and 3^rd^ QR: 828 hours) each during the 6-weeks. Compliance rates for each cohort were 68% (1^st^ and 3^rd^ QR: 41%-83%) equal to 16.3 hours/participant/day in the NL and 62% (1^st^ and 3^rd^ QR: 28%-82%) equal to 14.8 hours/participant/day in NAM (Fig 2).

### Attrition

In the NL, 13 participants (4% of all NL enrolled participants) did not contribute any data during the study period and were thus non-compliant. In NAM, this number was 135 (21% of all NAM enrolled participants). Additionally, 82 (27% of all enrolled) data-contributors in the NL became non-compliant during the study period. The primary known reasons (n = 47) were “Personal circumstances” (38%, n = 18) and “System too complex/System related issues” (34%, n = 16). For the NAM cohort, although reasons were unknown, this number was 89 (17% of all enrolled).

**Fig 3:**
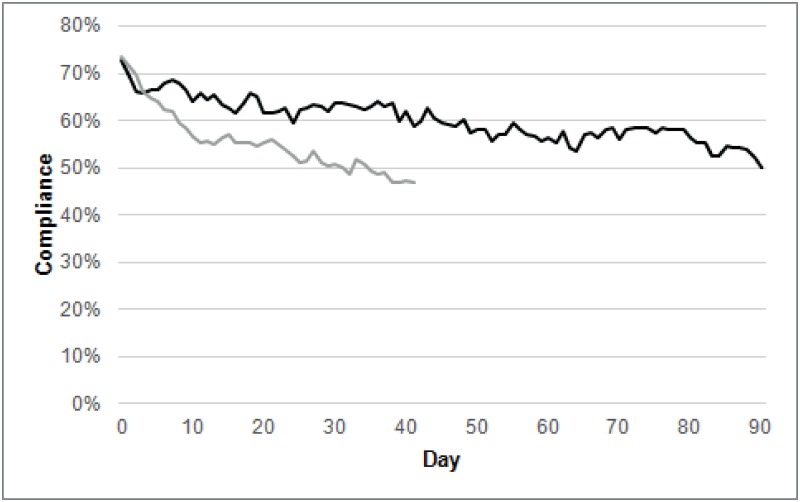
Attrition in compliance per day for NL (n = 291, black) and NAM participants (n = 514, gray) during the follow up period.

The attrition in the median percentage of sensor data collected daily varied between cohorts. In the NL, the attrition rate was 23% after 13-weeks’ study period. In the NAM, attrition was 27% after 6-weeks’ study period (Fig 3).

**Fig 4:**
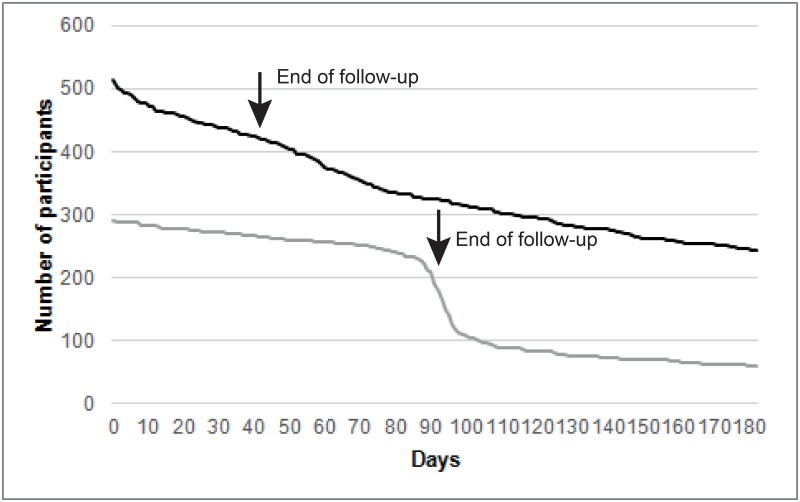
Number of participants actively collecting sensor data at the NL (gray) and NAM (black) cohorts during and after the follow-up period (total initial n = 805).

Attrition in participation was tracked during and beyond the compulsory study period for each cohort. he number of participants decreased rapidly after the end of the study period in the NL cohort. A more gradual attrition in participation occurred in the NAM cohort (Fig 4).

Ninety-six percent (n = 280) of data-contributors in the NL reported their medication through the app, while 78% (n = 404) did so in NAM. On average, data-contributors who used medication reports reported 351±217 medication intakes during the 13-week study period in the NL and 127±113 over 6-week study period in NAM. Both cohorts showed a low and non-exponential attrition in medication report, similar to the attrition showed in compliance with the accelerometer data (data not shown).

### System usability

**Fig 5:**
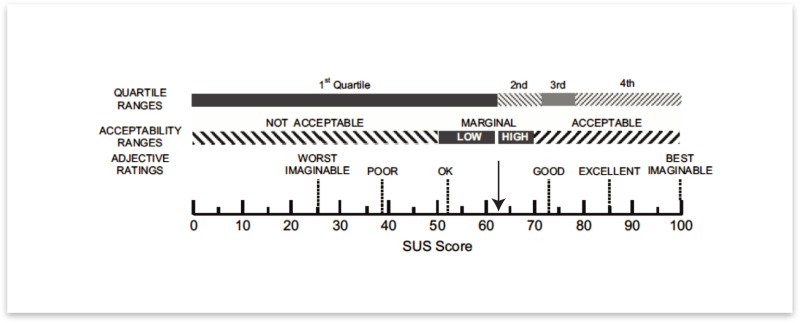
SUS scoring of the Fox Wearable Companion platform (smartwatch with smartphone app) as rated by participants.

In the NL cohort, 256 participants completed the System Usability Scale (response rate = 71.4%). The median score was 62.5 (1^st^ and 3^rd^ QT 47.5–72.5), which classifies the wearable platform in a category between “Ok” and “Good” (Fig 5).

### Factors related to compliance

After grouping all NL data-contributors into compliance groups, analysis reveals no significant differences in the distribution of demographics, clinical characteristics and ability to use a smartphone between these groups. However, Kruskal-Wallis analysis demonstrates that the System Usability score reported is significantly different between the groups (*χ*^2^ (2) = 32.014, p<0.001). The mean rank score is 84.8 for the low compliant group, 130.8 for the middle compliant group and 160.0 for the high compliant group, which indicates that participants in the high compliant group provided a higher usability score to the system.

For the NAM cohort, analysis shows that demographics and clinical characteristics between the three compliance groups was comparable, except for a trend regarding self-reported depression (*χ*^2^(2) = 6.397, p = .04). This result indicates that a slightly higher number of self-reported depressed patients are in the low compliant group (Table 3).

**Table 3:** Distribution of data-contributors’ characteristics and influence on compliance for the NL and NAM cohorts.

		The Netherlands				North America			
		Low compliance (n = 73)*	Middle compliance (n = 146)*	High compliance (n = 72)*	p-value	Low compliance (n = 128)*	Middle compliance (n = 256)*	High compliance (n = 129)*	p-value
Sex	Men	39 (71%)	84 (64%)	44 (65%)	.63^4^	64 (56%)	133 (54%)	62 (49%)	.50^4^
Age	≤50	7 (10%)	15 (10%)	7 (10%)	.95^4^	30 (26%)	49 (20%)	22 (17%)	.41^4^
	51–69	51 (70%)	103 (71%)	53 (75%)		67 (57%)	161 (65%)	81 (64%)	
	≥70	15 (20%)	27 (19%)	11 (16%)		20 (17%)	36 (15%)	24 (19%)	
Education Level	Low	11 (20%)	23 (17%)	17 (25%)	.68^4^	46 (41%)	84 (35%)	38 (31%)	.14^4^
	Middle	21 (38%)	45 (84%)	24 (35%)		43 (38%)	86 (35%)	57 (46%)	
	High	23 (42%)	51 (39%)	27 (40%)		24 (21%)	73 (30%)	29 (23%)	
Disease severity	0+1	15 (42%)	37 (28%)	21 (30%)	.55^5^	50 (44%)	121 (50%)	64 (52%)	.45
	2	15 (42%)	72 (54%)	40 (57%)		30 (26%)	66 (27%)	38 (31%)	
	3	6 (16%)	20 (15%)	8 (11%)		30 (26%)	48 (19%)	19 (15%)	
	4+5	0 (0%)	5 (4%)	1 (1%)		4 (4%)	7 (3%)	2 (2%)	
Depression^1^	No (<6)	36(100%)	135 (98%)	67 (94%)	.28^5^	74 (63.2%)	172 (70%)	99 (78%)	.04^4^
Cognitive impairment^2^	No (>26)	16 (47%)	74 (56%)	34 (50%)	.58^4^	-	-	-	-
Level of independency^3^	≤70	5 (14%)	21 (16%)	10 (15%)	.16^4^	-	-	-	-
	71–80	13 (36%)	30 (23%)	8 (12%)		-	-	-	
	81–90	13 (36%)	61 (46%)	35 (52%)		-	-	-	
	≥91	5 (14%)	21 (16%)	15 (22%)		-	-	-	
How easy is for you to use a smartphone?	Very easy	12 (19%)	33 (24%)	14 (21%)	.06^5^	-	-	-	-
	Easy	27 (44%)	60 (44%)	30 (44%)		-	-	-	
	Neither easy nor difficult	14 (23%)	27 (20%)	23 (34%)		-	-	-	
	Difficult	5 (8%)	14 (10%)	1 (2%)			-	-	
	Very difficult	4 (6%)	2 (2%)	(0%)		-	-	-	-
MDS-UPDRS^6^ (median)		49	55	48	.55	-	-	-	-
SUS^6^ (median)		50	65	70	<0.001	-	-	-	-

*Missing values varied across variables.

Red is significant at .05; green is significant at .001.

SES: Socioeconomic status; Disease severity: assessed with Hoehn and Yarh stages at the NL cohort and estimated from self-reported at NAM cohort; GDS: Geriatric Depression Scale; MoCA: Montreal Cognitive Assessment, MDS-UPDRS: Movement Disorders Society—Unified Parkinson’s Disease Rating Scale, SUS: System Usability Scale.

^1^-Depression was assessed with the Geriatric Depression Scale at NL and self-reported on NAM;

^2^-Cognitive impairment was assessed using the Montreal Cognitive Assessment;

^3^-Independency level was measured by Modified Schwab and England Activities of Daily Living Scale;

^4^ –Pearson Chi-Square;

^5^ –Fisher’s Exact Test;

^6^-Kruskal-Wallis

## Discussion

This study assessed the feasibility of using a wearable platform for long-term data collection in a large sample of PD patients. We focused on: recruitment success, attrition rates, compliance and system usability. Enrolment rate was 88% (n = 304) in the NL and 51% (n = 649) in NAM. Nearly 85% of all enrolled participants contributed sensor data during the study period. Median compliance rate was 68% (16.3 hours/participant/day) in the NL, and 62% (14.8 hours/participant/day) in NAM. The rate of accelerometer data collected each day declined 23% in the NL after 13-weeks of study period, and 27% in NAM after 6-weeks of study period. The distribution of demographics, clinical characteristics and ability to use a smartphone did not differ across compliance groups in the NL, but System Usability score did differ. For the NAM, the distribution of demographics and clinical characteristics between the compliance groups was comparable, except for self-reported depression status.

The high compliance in this study shows that it is feasible for people with PD to use this wearable platform in a real-world environment for many months. Although the feasibility of using consumer wearable sensors to monitor PD symptoms has been previous reported[9, 13, 17, 18, 31, 35], this is the first rigorous observational study to investigate the feasibility of a wearable platform comprising a smartwatch combined with a smartphone in such a large patient group (the largest prior study included only 40 patients). Additionally, the small differences in study protocols across cohorts allowed us to observe the impact of varying usage instructions on compliance. Comparing the feasibility results obtained in this study to other studies, where either mobile apps were used in large cohorts[36] or e-health technologies were use[37, 38] d, we achieved a high compliance together with small and non-exponentially decreasing attrition rate, even though exponential decrease in compliance is the norm in these sort of studies[33].

This unusually high compliance rate may be attributed to the “*passive”* data collection. In this case, little or no interaction with the technology is required in order to collect sensor data. Participants using the Parkinson@Home wearable platform, other than reporting their medication intake when reminded by the alarm (which was widely perceived as a service, instead of a burden), did not need to interact actively with the smartphone or smartwatch. In another similar smartphone-based study where “*active”*, “*task-based*” monitoring was used (that is, where participants needed to perform certain specific tasks, at regular intervals prompted by the platform)[36], a more typical high and exponential attrition rate was observed. While it is difficult to draw firm conclusions from this comparison (because the two platforms are somewhat different), we suspect that periodic and long interaction by users may increase attrition, leading to attrition rates seen in paper-based diaries[39]. The low and non-exponential attrition seen in the medication reports, a quick and less burdensome task, strengthened this conclusion. Thus, passive monitoring, where little to no interaction with the technology is required, may lead to better overall compliance rates.

Despite the potential influence of age, gender and PD-related impairment (i.e. physical or cognitive) on compliance, our results showed that overall disease severity, MDS-UPDRS scores, independency level or cognitive impairment, did not influence compliance, which suggests that this platform could be used by most PD patients. The unique design of the Parkinson@home study can partially explain this result. The presence of a personalized support centre, which was previously described as an effective strategy to improve retention of participants[33], may have increased patients’ confidence in using the system and have compensated for any disease-related difficulties.

Moreover, the “pro-active” support model, with scheduled calls to participants who showed signs of low compliance, may have boosted compliance by providing a quick resolution of technical interruptions, and addressed any apathy towards participation caused by technology difficulties. This support is even more important because compliance is compromised in participants that reported low System Usability scores. Therefore, in order to achieve high compliance while using smartphone/smartwatch wearable platforms to measure PD related symptoms at home, it is beneficial to: (1) improve the platform’s usability, (2) reduce the number of technical issues, and (3) run a personalized support centre that can provide guidance to deal with possible technology related issues that participants may encounter.

### Limitations

The Parkinson@Home study did have a few limitations. First, this is one of the first large-scale cohort studies using consumer wearable sensors in PD, with a long study period duration (i.e. up to 13 weeks). However, the study sample consisted only of PD patients that possessed a smartphone, thus introducing a possible selection bias, e.g. towards more highly educated subjects. Although smartphone penetration in the NL and NAM is high[40, 41], participants may not reflect the majority of PD patients living in the Netherlands, North America, or elsewhere in the world. Furthermore, when compared to the general PD population[42], participants were mainly young with a mild disease impairment and with some degree of cognitive impaired. Even though these variables showed no obvious influence on compliance, a more impaired population may need more personal support in order to maintain compliance. Future studies should aim for a more stratified population in order to further confirm the lack of influence across the full range of disease severity in the compliance with wearable sensors among PD patients. Second, the present results only apply to the use of two specific consumer grade devices (i.e. smartphone and smartwatch). Although consumer grade devices bring potential advantage over the use of dedicated medical devices, it is unknown whether our promising feasibility results would generalize to dedicated medical devices which are often more expensive and less user-friendly.

## Conclusion

In conclusion, the Parkinson@home trial showed that it is feasible to deploy a technology platform consisting of consumer-grade wearable and mobile devices for long-term data collection in a large and geographically diverse PD population. Importantly, compliance was comparable for patients with a range of backgrounds, including men and women, different ages, and some variations in disease severity. These findings suggest that wearables may offer a promising approach to overcome the limitations in monitoring disease status and progression of mildly impaired PD patients in a real-life environment. The platform here used is a promising and practical approach to capturing large amounts of sensor data from many participants by passive means, without much need for interaction with the technology. In the future, these properties may position sensor technologies as effective tools for monitoring PD and the “lived experience” of PD patients.

## Supporting information

S1 File Scale created by the researchers to measure ability to use a smartphone. (TIF)
