# Supplementary figures and images for: Feasibility of large-scale deployment of multiple wearable sensors in Parkinson's disease
Source: PLoS One. 2017 Dec 20;12(12):e0189161. doi: 10.1371/journal.pone.0189161 (PMC5738046; doi:10.1371/journal.pone.0189161)

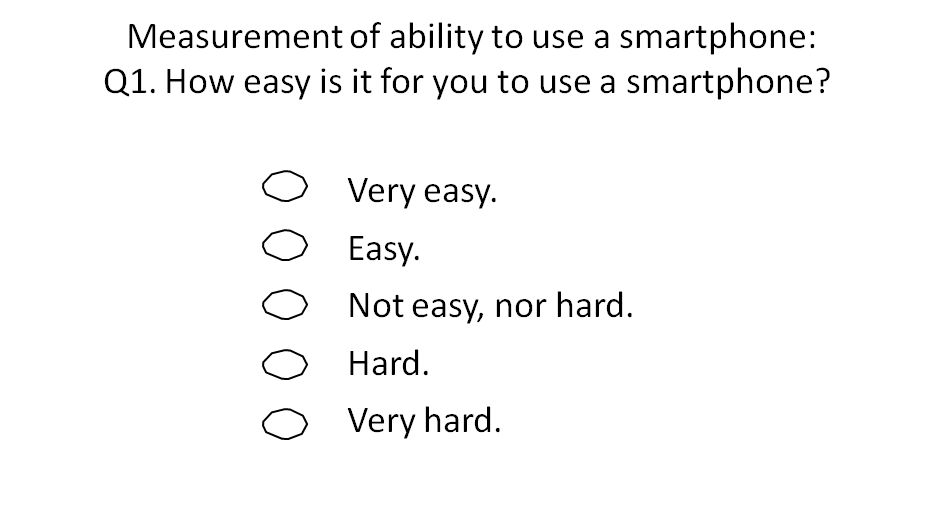

Supplement: S1 File — (TIF) [file pone.0189161.s001.tif]
